# Beyond histones: the elusive substrates of chromatin regulators

**DOI:** 10.1101/gad.351969.124

**Published:** 2024-05-01

**Authors:** Mattias Mannervik

**Affiliations:** Department of Molecular Biosciences, The Wenner-Gren Institute, Stockholm University, Stockholm 10691, Sweden

**Keywords:** chromatin, *Drosophila melanogaster*, epigenetics, gene expression, genomics, H4K20me1, L(3)mbt, Set8

## Abstract

Gene transcription is intimately linked to chromatin state and histone modifications. However, the enzymes mediating these post-translational modifications have many additional, nonhistone substrates, making it difficult to ascribe the most relevant modification. In this issue of *Genes & Development*, Crain and colleagues (doi:10.1101/gad.351698.124) have combined a powerful histone replacement system with mutational analysis of a chromatin regulator and a chromatin reader in *Drosophila melanogaster*. Importantly, they discovered that genes controlled by the histone 4 lysine 20 (H4K20) methyltransferase Set8 and the protein recognizing H4K20 monomethylation, L(3)mbt, differ substantially from those affected by mutation of H4K20 itself. This demonstrates that H4K20 is not the key substrate for Set8 but that methylation of other, unidentified proteins mediates its effects on transcription.

## Abstract (precis)

In this Outlook, Mannervik discusses a study in this issue of *Genes & Development* by Crain et al. that shows that genes controlled by the histone 4 lysine 20 (H4K20) methyltransferase Set8 and the protein recognizing H4K20 monomethylation, L(3)mbt, differ substantially from those affected by mutation of H4K20 itself.

Transcription is essential for cell specification and cell state transitions and is closely linked to chromatin state. A number of histone modifications are associated with most transcriptionally active genes, whereas other histone modifications lead to gene silencing. The enzymes mediating these post-translational modifications are frequently mutated in cancer and other disorders, implicating chromatin state in disease. However, chromatin regulators also have many nonhistone substrates as well as nonenzymatic functions. It is therefore necessary to elucidate which molecular processes and substrates are critical for chromatin regulator function if we are to explain their involvement in disease.

Histones are needed in high quantities during each cell division to assemble additional nucleosomes on the newly replicated DNA. To ensure this high supply, histones are encoded by multicopy genes. For example, there are 16 histone H4 genes in humans located at three different chromosomal locations (Seal et al. 2022). Consequently, it has been challenging to conduct classical genetic analysis of histones. However, the advent of CRISPR/Cas9 technology has opened avenues for mutating all histone genes in mammalian cells (Sankar et al. 2022). Nevertheless, to investigate the role of histone amino acid substitutions in a whole animal, *Drosophila melanogaster* remains the sole option. In this organism, all replication-dependent histone genes are located in a single chromosomal cluster. This cluster contains ∼100 histone gene units, each comprising the five histone genes H1, H2A, H2B, H3, and H4. Remarkably, a fly in which this cluster has been deleted can be rescued to viability with only 12 copies of transgenic histone gene units (Günesdogan et al. 2010). Individual amino acids can be mutated in all 12 transgenic copies, thereby creating an animal that expresses only mutant histones.

Using this histone replacement system, it was previously shown that substitution of H3 lysine 27 for arginine (H3K27R) results in derepression of *Hox* genes, a classical Polycomb phenotype (Pengelly et al. 2013; McKay et al. 2015). This demonstrates that H3K27 is the relevant and critical substrate for Polycomb-mediated heterochromatin silencing, involving H3K27 trimethylation (H3K27me3) by the methyltransferase Enhancer of zeste, the homolog of mammalian EZH2.

While H3K27 methylation results in heterochromatin formation and gene silencing, methylation of H4K20 is a more enigmatic histone modification (for review, see Beck et al. 2012). H4K20 monomethylation (H4K20me1) has been linked to both gene activation and gene silencing. H4K20me1 is found on the inactive X chromosome in mammals and may promote chromatin compaction. However, the overall genomic distribution of H4K20me1 shows that it is linked to active gene transcription. The enzyme responsible for H4K20me1 is alternatively known as PR-Set7, KMT5A, SETD8, or Set8. Loss of this protein results in embryonic lethality in mice and lethality at the third instar larval stage in *Drosophila*.

In a previous study, Crain et al. (2022) showed that the developmental phenotypes of *Drosophila* Set8 and H4K20 mutants differ. In this issue of *Genes & Development* Crain et al. (2024), report the results from RNA-seq experiments, revealing substantial differences in the transcriptomes of Set8 and H4K20 mutants. This clearly demonstrates that regulation of gene expression by Set8 is not mediated via H4K20 methylation. H4K20me1 is recognized by the protein lethal (3) malignant brain tumor [L(3)mbt], which is associated with transcriptional repression through E2F/Rb and with processes that affect the cell cycle. How can the links of H4K20me1 to active genes and of L(3)mbt to gene repression be reconciled? Crain et al. (2024) found that although L(3)mbt associates with many genes containing H4K20me1, it primarily occupies promoter regions, whereas H4K20me1 is found throughout transcribed gene loci. Furthermore, they discovered that the association of L(3)mbt with promoter regions was not decreased but rather increased in animals that lack H4K20 methylation due to substitution of H4K20 for arginine. Taken together, this shows that L(3)mbt promoter occupancy is not dependent on H4K20me1 and distinguishes L(3)mbt-mediated repression from H4K20me1.

Intriguingly, substitution of H4K20 for alanine (H4K20A) or arginine (H4K20R) elicits different phenotypes, although methylation of H4K20 is prevented by both substitutions. Approximately 20% of H4K20A animals survive, whereas all H4K20R mutants die. This is reflected in gene expression changes, where H4K20A mutants misregulate only 90 genes, while >1200 genes are not properly expressed in H4K20R animals. It is noteworthy that in both cases, the large majority of affected genes lack or have very low levels of H4K20me1 in wild-type animals. These results establish that H4K20me1 does not generally influence gene expression.

What, then, could be the difference between H4K20A and H4K20R animals? This brings us back to the surprising finding that L(3)mbt binds more strongly, not less, to chromatin in H4K20R animals. Additional experiments by Crain et al. (2024) demonstrated that both Set8 and L(3)mbt proteins bind more strongly to H4K20R recombinant nucleosomes than to nucleosomes with a normal, wild-type H4. It is therefore possible that Set8 and L(3)mbt become trapped on H4K20R chromatin, which disrupts their normal function. Consistent with this idea, some of the gene expression changes in H4K20R animals overlap those of Set8 and L(3)mbt mutants but not those in H4K20A animals. Thus, histone amino acid substitutions may influence binding by chromatin regulators independent of their impact on post-translational modifications. This partly resembles the effects of “oncohistones,” where missense mutations in one histone gene copy can result in cancer. It is believed that the mutant histones in such instances dominantly inhibit the corresponding histone methyltransferase, which affects the transcriptome and epigenome in a way that promotes tumor formation (for review, see Sahu and Lu 2022). In these cases, however, the substitution is commonly from lysine to methionine (e.g., H3K27M), and there is no evidence that H4K20R dominantly affects Set8.

If H4K20me1 does not affect gene expression, what is its function? Both H4K20me1 and Set8 levels fluctuate during the cell cycle and have been implicated in cell cycle progression, DNA replication, and DNA damage (Beck et al. 2012). Exactly how H4K20me1 and Set8 affect DNA replication is not clear, but DNA damage in Set8 mutants accumulates only if cells enter S phase, suggesting that these processes are linked. Crain et al. (2024) observed that while Set8 mutant cells fail to proliferate, H4K20 mutant cells can divide. Thus, at least some of the cell cycle phenotypes in Set8 mutants are not mediated through H4K20me1.

Since Set8 does not affect gene expression and cell cycle progression through H420 methylation, it either methylates other proteins or has catalytic-independent functions. Crain et al. (2024) showed that a Set8 mutant with impaired catalytic activity misregulates much fewer genes than a Set8-null mutant. This indicates that nonenzymatic functions are most important, but that methylation of proteins other than histone H4 is also involved in Set8-mediated gene regulation. A few nonhistone Set8 substrates, including p53 and PCNA, have been identified (for review, see Milite et al. 2016). However, the critical target(s) in Set8-mediated gene regulation remain(s) unknown. Identifying catalytic-independent functions and the elusive nonhistone substrates of Set8 and other chromatin regulators will be critical for understanding their role in disease.
